# Cost-effectiveness of an exercise intervention program in perimenopausal women: the Fitness League Against MENopause COst (FLAMENCO) randomized controlled trial

**DOI:** 10.1186/s12889-015-1868-1

**Published:** 2015-06-17

**Authors:** Ana Carbonell-Baeza, Alberto Soriano-Maldonado, Francisco Javier Gallo, María Puerto López del Amo, Pilar Ruiz-Cabello, Ana Andrade, Milkana Borges-Cosic, Antonio Rubén Peces-Rama, Zuzana Spacírová, Inmaculada C. Álvarez-Gallardo, Leticia García-Mochón, Víctor Segura-Jiménez, Fernando Estévez-López, Daniel Camiletti-Moirón, Jose Jesús Martín-Martín, Pilar Aranda, Manuel Delgado-Fernández, Virginia A. Aparicio

**Affiliations:** Department of Physical Education, Faculty of Education Science, University of Cádiz, Cádiz, Spain; Department of Physical Education and Sport, Faculty of Sport Sciences, University of Granada, Granada, Spain; Zaidín Sur healthcare centre and Department of Medicine, Faculty of Medicine, University of Granada, Granada, Spain; Department of Applied Economics, Faculty of Economics, University of Granada, Granada, Spain; Andalusian School of Public Health, Granada, Spain; Department of Clinical and Health Psychology, Utrecht University, Utrecht, The Netherlands; Department of Physiology, Faculty of Pharmacy, Faculty of Sport Sciences, and Institute of Nutrition and Food Technology, University of Granada, Granada, Spain; Department of Public and Occupational Health, EMGO+ Institute for Health and Care Research, VU University Medical Centre, Amsterdam, The Netherlands

**Keywords:** Cost-effectiveness, Health status, Quality of life, Physical activity, Physical fitness, Primary care

## Abstract

**Background:**

The high prevalence of women that do not reach the recommended level of physical activity is worrisome. A sedentary lifestyle has negative consequences on health status and increases health care costs. The main objective of this project is to assess the cost-effectiveness of a primary care-based exercise intervention in perimenopausal women.

**Methods/Design:**

The present study is a Randomized Controlled Trial.

A total of 150 eligible women will be recruited and randomly assigned to either a 16-week exercise intervention (3 sessions/week), or to usual care (control) group.

The primary outcome measure is the incremental cost-effectiveness ratio. The secondary outcome measures are: i) socio-demographic and clinical information; ii) body composition; iii) dietary patterns; iv) glycaemic and lipid profile; v) physical fitness; vi) physical activity and sedentary behaviour; vii) sleep quality; viii) quality of life, mental health and positive health; ix) menopause symptoms. All outcomes will be assessed at baseline and post intervention. The data will be analysed on an intention-to-treat basis and per protocol. In addition, we will conduct a cost effectiveness analysis from a health system perspective.

**Discussion:**

The intervention designed is feasible and if it proves to be clinically and cost effective, it can be easily transferred to other similar contexts. Consequently, the findings of this project might help the Health Systems to identify strategies for primary prevention and health promotion as well as to reduce health care requirements and costs.

**Trial registration:**

ClinicalTrials.gov Identifier: NCT02358109. Date of registration: 05/02/2015

## Background

Physical inactivity is a pandemic and a leading risk factor for mortality [1]. Worldwide, 31.1 % of adults are physically inactive [2]. Inactivity rises with age, is higher in women than in men, and is increased in high-income countries [2]. In Spain, 50.2 % of adults are physically inactive (47.4 and 53.1 % in men and women, respectively) [2]. The elevated percentage of women who do not reach recommended level of physical activity is worrisome. A recent study in Spain [3] found that women aged from 41 to 50 years emerged as the groups with a greater likelihood of being sedentary in their leisure time. This physical inactivity-derived problem can be especially relevant during the perimenopausal period. Beyond the negative effects of physical inactivity [1], menopause increases cardiometabolic risk factors due to the significant decline in the estrogen levels [4, 5]. Moreover, menopause-related testosterone predominance appears to be implicated as a key hormonal change associated with the incidence of metabolic syndrome, independent of aging and other standard cardiovascular disease (CVD) risk factors [6]. Regular physical activity is associated with decreased risk of CVD and type 2 diabetes in middle aged women, independently of their menopause status [7]. Similarly, some studies observed that postmenopausal women with high physical activity levels had a more favourable CVD risk profile than their physically inactive peers [8, 9]. Additionally, higher physical activity is directly associated with better health-related quality of life [10, 11]. Indeed, exercise interventions can improve sleep quality [12] and depression [13] in middle adult women. However, the role of physical activity in clinical practice remains undervalued despite increasing evidence supporting its protective effects and the economic burden associated with a sedentary lifestyle [1].

National Health expenditures in Spain have increased from 7.2 % of the gross domestic product (GDP) in 2001 to 9.3 % in 2011 [14]. The pharmacological treatment costs are specially increasing in European countries [14]. In Spain, pharmacological costs represent 1.6 % of the GDP, and 17.4 % of the National Health Expenditures [14]. Previous studies have shown that regular physical activity reduces health care requirements and thereby leads to significant savings in health-care costs [15, 16].

In 2005, the Spanish Ministry for Health and Consumption designed the Strategy for Nutrition, Physical Activity and Prevention of Obesity (NAOS) with the aim of promoting a healthy lifestyle among the general population. Additionally, in May 2009 the Spanish National Sports Council launched the Integral Plan on Physical Activity and Sport (A + D Plan). This plan included a specific program focused on women, with the objective of increasing their physical activity levels. In this context, we designed the Fitness League Against MENopause COst (FLAMENCO) project, a randomized controlled trial to investigate the cost-effectiveness of an exercise intervention in primary care settings in middle aged women.

The primary aim of the FLAMENCO project is to assess the cost-effectiveness of a primary care-based exercise intervention program (16 weeks) in perimenopausal women. In addition, we will address the following specific objectives: i) to determine the associations between physical activity, sedentary behaviour and physical fitness with CVD biomarkers, body composition, dietary patterns, glycaemic and lipid profile, sleep quality, quality of life, mental health, positive health and menopause symptoms; and ii) to study the associations between the above mentioned variables with pharmacological and health service costs.

The purpose of this methodological article is to describe the study design, procedures and methods that will be used in the “FLAMENCO project”.

## Methods/Design

The present study is a Randomized Controlled Trial (RCT) (registration number: NCT02358109). The study protocol has been reviewed and approved by the Ethics Committee for Research Involving Human Subjects at the University of Granada. The participants will have to provide a written informed consent before taking part in the study, which will be conducted in accordance with the CONSORT (Consolidated Standards of Reporting Trials) statement.

### Participants and recruitment process

Women will be recruited through primary care centres from Granada (Southern Spain). Medical staff will recruit possible candidates for participation during consultations at the primary care centres. Furthermore, press releases will be published in local newspaper and on social media (radio, internet, etc.). Moreover, information leaflets will be distributed in primary care centres and informative meetings with potential participants will be carried out.

**Fig. 1:**
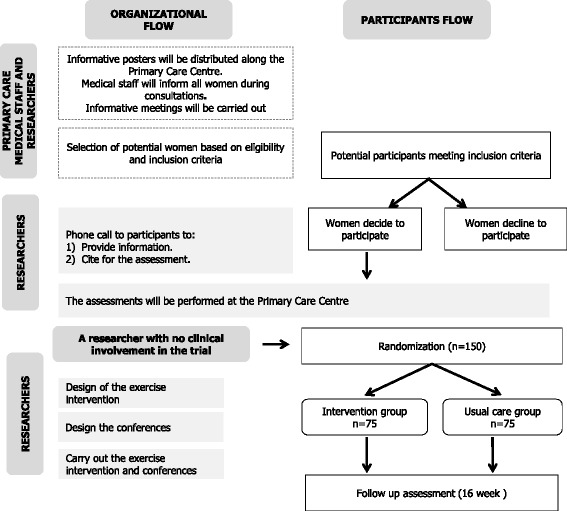
The organizational and participants flow

A screening of all candidates will be performed. The inclusion and exclusion criteria are shown in Table 1. The organizational and participants flow is presented in Fig. 1.

**Table 1:** Inclusion and exclusion criteria in the FLAMENCO project

Inclusion criteria
- Age: 45–60 years old.
- Not to have severe somatic or psychiatric disorders, or diseases that prevent physical exercise (Answer “no” to all questions on the PAR-Q).
- Not to be engaged in regular physical activity >20 min on >3 days/week in the last three months.
- To be able to ambulate without assistance.
- To be able to communicate.
- Informed consent: To be capable and willing to provide informed consent.
Exclusion criteria
- Acute or terminal illness.
- Myocardial infarction in the past 3 months.
- Unstable cardiovascular disease or other medical condition.
- Upper or lower extremity fracture in the past 3 months.
- Unwillingness to either complete the study requirements or to be randomised into control or intervention group.
- Presence of neuromuscular disease or drugs affecting neuromuscular function.

PAR-Q: Physical Activity Readiness Questionnaire

### Sample size

The number of participants to be included in the study was calculated on the basis of the change in quality of life as assessed with the *EuroQol 5D (*EQ-5D-5 L). To the best of our knowledge, there is no available data regarding group differences in changes in quality of life following any type of intervention in middle aged women. Therefore, we assumed a difference of 0.07 units as clinically meaningful based on previous observations from our group (unpublished observations). Therefore, a total of 124 participants (62 per group) is needed to detect a mean group difference of 0.07 and a standard deviation of 0.12 in the total EQ-5D score with a power of 90 % and α of 0.05. However, we will attempt to exceed this sample size to allow for withdrawals. Assuming a maximum lost-to-follow up of 20 %, a minimum of 75 participants per group will be recruited (*n* = 150).

### Randomization and blinding

After baseline assessments, all participants will be randomized to either exercise intervention or control (non-exercise) group. A computer generated simple randomization sequence will be created before participants will be enrolled, to allocate participants to either the exercise intervention or control group (1:1). The randomization sequence will be prepared by a member of the research team with no clinical involvement in the trial. The allocation will be concealed in a password protected computer file. Whereas the participants will be aware of their group allocation, outcome assessors and data analysts will be blinded to the allocation.

### Interventions

The participants randomly assigned to the usual care (control) group will receive general advices from primary care centre medical staff about the positive effects of physical activity. The researchers will give 4 conferences (1 per month) explaining the benefits of exercise for longevity, prevention and treatment of diseases, the benefits of the Mediterranean diet, nutritional education, ergonomic advises, exercise to perform at home (e.g. stretching, strength training) and strategies to increase their daily physical activity levels.

The exercise intervention will be performed in four groups so that each group will comprise 15–20 participants. The groups will train 3 days/week (60 min per session) for a 16-week period at the primary care centre of Granada. The exercise intervention will meet the training standards of the American College of Sports Medicine [17] for adults. The exercise sessions will be designed, carefully supervised, guided and instructed by qualified exercise professionals.

Each exercise session will include a 10 min warm-up period with walks and mobility exercises, followed by 40 min of a main part which will vary across week days (i.e. 3 different models of session). Sessions will finish with a 10 min cool-down period of stretching and relaxation exercises. The three model sessions will be: (i) First session of the week will involve circuit training including resistance exercises in a stepped progression during the program. Resistance circuit training will include 2–3 set of 6–10 exercises. The participants will perform 10–20 repetitions of multi-joint exercises and/or single joint exercises targeting major muscle groups such as biceps curls, triceps extensions, arm side lifts, shoulder elevations, chest press, lateral leg elevations, stands up from seated position, lunge, sideways lunge and step-up/step-down, squads, lower back extensions, abdominal crunch/curl-up and calf raises among others. Participants will carry out bodyweight exercises at the beginning of the program and, if it is necessary, free weights (0.5–3 kg barbells) and resistance bands will be used to reach the optimal intensity. The load will be gradually increased and each participant will individualize her effort to reach the intensity designed for each session; (ii) Second session of the week will include balance-oriented activities (position changes, monopodal and bipodal stances, backwards walks, …) and dancing (aerobic exercises); (iii) Third session of the week will combine aerobic, resistance strength and coordination exercises.

Heart rate will be measured with heart rate monitors (Polar Electro OY, Finland) to control the intensity of the sessions. One third of the participants in the intervention group will wear heart rate monitors in 1/3 of the sessions, both randomly selected. We will monitor the ratings of perceived exertion (RPE) using the Borg 6-20 RPE scale [18] during all the sessions. Intensity (expressed as RPE) will be expected to range from 12 to 16. The exercise volume and intensity programmed is shown in Table 2.

**Table 2:** Exercise intervention

Sesion structure	Content								
Warm-up 10’ minutes	Joint mobility and different walk modalities								
Conditioning 40 min	Week	1-2	3-4	5-6	7-8	9-10	11-12	13-14	15-16
	Intensity RPE	12-13	12-13	13-14	13-14	14-15	14-15	15-16	15-16
	Monday								
	Resistance training circuit*	6 exercises	8 exercises	8 exercises	8 exercises	8 exercises	8 exercises	10 exercises	10 exercises
	Set x repetitions	2 × 15	3 × 15	3 × 18	3 × 20	3 × 18	3 × 15	3 × 12	3 × 10
	Wednesday	Choreographies and aerobic exercises							
	Friday	5 exercises	6 exercises	6 exercises	6 exercises	7 exercises	7 exercises	8 exercises	8 exercises
	Resistance exercises (RE)^a^ and aerobic exercises (AE)^b^	2RE + 1AE + 2RE	3RE + 1AE + 2RE	3RE + 1AE+ 2RE	3RE + 1AE+ 2RE	2RE + 1AE + 2RE +1AE + 1RE	2RE + 1AE+ 2RE + 1AE + 1RE	2RE + 1AE + 2RE +1AE +2RE	2RE +1AE + 2RE +1AE + 2RE
Cool-down 10 min	Stretching and relaxation exercises								

RPE, rating of perceived exertion; *The circuit is composed of trunk, upper and lower extremities exercises. Body-weight, free weights (0.5–3 kg barbells) and resistance bands exercises. The load will be gradually and individualized increased for each participant to reach the intensity designed for each session; ^a^The set and repetitions in resistance exercise will be the same than the resistance circuit training;^b^ The aerobic exercise will have a duration between 2–4 min

To maximize adherence, several strategies will be implemented including music in all sessions, individualized attention during the intervention sessions, and telephone calls following non-attendance. The researchers will control and register the presence of adverse event during and between classes.

### Primary and secondary outcomes

#### Primary outcomes

The Incremental Cost Effectiveness Ratio (ICER) [19]. For each group (intervention and control) an average of cost and health effects will be calculated. The measurement of health effects is defined as the Quality Adjusted Life Years (QALYs). The QALYs are calculated multiplying the years of life by the participants’ quality of life. The participants’ quality of life will be assessed by the EQ-5D-5 L questionnaire. The ICER will be calculated dividing the difference between the average costs of both groups by the difference in mean QALYs gained in both groups [19].

$$ \mathrm{ICER}=\frac{\mathrm{Cos}{\mathrm{t}}_{\mathrm{Intervention}}-\mathrm{C}\mathrm{o}\mathrm{s}{\mathrm{t}}_{\mathrm{Control}}}{\mathrm{QAL}{\mathrm{Y}}_{\mathrm{Intervention}}-\mathrm{Q}\mathrm{A}\mathrm{L}{\mathrm{Y}}_{\mathrm{Control}}} $$

### Secondary outcomes

#### Socio-demographic and clinical information

Demographic and other health information will be collected using a questionnaire. This anamnesis will include questions regarding smoking and alcohol habits, history of illness (as osteoporosis, CVD, hypertension, diabetes), menopause status, indicators of socio-economic status (such as personal and household income, education level…), marital status, and number of children. The pharmacology register as well as the number and reason of medical visits will be consulted by the primary care medical staff in collaboration with members of the research team through the medical database.

Systolic and diastolic blood pressure, as well as resting heart rate, will be measured after 5 min of rest, on 2 separate occasions (with 2 min between trials), with the person seated (Omron Health Care Europe B.V. Hoolddorp). The lowest value of the two trials will be selected for the analysis.

#### Body composition

Lean, fat and bone mass of the whole body will be measured using a dual-energy x-ray absorptiometry (DXA) device (Hologic Discovery QDR, Nasdaq: HOLX). Height (cm) will be measured using a stadiometer (Seca 22, Hamburg, Germany). Waist circumference (cm) will be assessed at the middle point between the ribs and the ileac crest, with the participant standing (Harpenden anthropometric tape, Holtain Ltd).

#### Dietary patterns

The *Mediterranean Diet Score* [20] is an index created to evaluate the degree of adherence to the traditional Mediterranean dietary pattern. It consists of 11 items (non-refined cereals, potatoes, fruits, vegetables, legumes, fish, olive oil, red meat and derivate, poultry, full fat dairy products and alcohol), which scores ranging from 0–5 based on frequency of consumption. Thus, the total score ranges from 0–55, with higher scores indicating greater adhesion to the Mediterranean dietary pattern.

The *Food Frequency questionnaire* [21] consists on a list of 78 foods on which participants will be asked to indicate the frequency of consumption (never or number of times per day, per week, per month or per year).

#### Glycaemic and lipid profile

Venous blood samples will be collected in two vacuum tubes in standardized fasting conditions at 8–9 a.m. in the primary care centre and transported to the laboratory for subsequent analysis. One of these vacuums will contain EDTA/K3 to determine blood cells count, blood haemoglobin concentration and haematocrit. Plasma total cholesterol, high-density lipoprotein cholesterol, low-density lipoprotein cholesterol, triglycerides, glucose, insulin, albumin, glycosylated haemoglobin and thyroid-stimulating hormone levels will be assessed with standard methods using an autoanalyzer (Hitachi-Roche p800, F. Hoffmann-La Roche Ltd. Switzerland).

#### Physical fitness

Participants’ physical fitness status will be assessed by means of the following tests:

##### Cardiorespiratory fitness

The *modified Bruce protocol* [22, 23] will be performed to estimate maximal oxygen uptake (VO_2max_), and will be used as measure of cardiorespiratory fitness. The test will consist of 5 increasing workload stages of 3 min each (stage 1: 2.7 km/h and 10 % inclination; stage 2: 4 km/h and 12 % inclination; stage 3: 5.5 km/h and 14 % inclination; stage 4: 6.8 km/h and 16 % inclination; stage 5: 8 km/h and 18 % inclination). The test will conclude when the 85 % of the individual’s heart rate reserve (HRR) is accomplished. VO_2max_ will be estimated with the formula by Bruce et al. [23]: VO_2max_ = 6.70 – 2.82*2 + 0.056*duration of the test (s).

We will additionally perform the *6-min walk test*, which measures the maximum distance (in meters) each participant can walk in 6 min along a 45.7 m rectangular course [24].

##### Lower-body muscular strength

The *30-s chair stand test* involves counting the number of times within 30 s that the participant can rise to a full stand from a seated position with back straight and feet flat on the floor, without pushing off with the arms. The participants will perform one trial after familiarization [24].

##### Upper-body muscular strength

The *arm curl test* involves determining the number of times a hand weight (2.3 kg for women) can be curled through a full range of motion in 30 s seconds [24]. Additionally, *handgrip strength test* will be measured using a digital dynamometer (TKK 5101 Grip-D; Takey, Tokyo, Japan) as described elsewhere [25]. The participants will perform (alternately with both hands) these tests twice. The best value of 2 trials for each hand will be chosen and the average of both hands will be used in the analyses.

##### Lower-body flexibility

The *sit and reach test* required the use of the sit-and-reach standardized box with a slide ruler attached to the top [26]. The participant will be required to sit with knees straight and legs together, and feet placed against the box. The participant slowly will reach forward as far as possible [26]. The final position that the participant reach in centimetres will be the test score. The best score of two attempts will be recorded. Additionally, the *back saver sit and reach test* will be carried out in the same sit-and-reach standard box described above. The participant will be required to sit with the untested leg bent at the knee and the tested leg with the knee straight and feet place against the box. The scoring is the same as the sit and reach test but only one leg is evaluated at a time. The best score of two attempts for each leg in centimetres will be recorded and the average of both legs will be used in the analyses.

##### Upper-body flexibility

The *back scratch test*, a measure of overall shoulder range of motion, involves measuring the distance between (or overlap of) the middle fingers behind the back with a ruler [24]. The best score of two attempts for each arm in centimetres will be recorded and the average of both arms will be used in the analyses.

##### Motor agility/dynamic balance

The *timed up and go test* [27] will be performed to assess the dynamic balance. The participant will be seated in a chair with arms and trunk supported. The participant will be instructed to stand up on the word “go” and walk 3 meters in a straight line, turned 180°, walk back to the chair and sit down again in the chair. One familiarization trial will be undertaken. After 1-min rest, the test will be performed twice separated by 1-min. The time from the start until the participant sit down in the chair with back support will be measured and the best of the 2 attempts will be used.

##### Self-perceived physical fitness

The *International Fitness Scale* [28] consists of five Likert scale questions asking how participants perceive their overall fitness, cardio-respiratory fitness, muscular strength, speed-agility and flexibility (“very poor”, “poor”, “average”, “good” and “very good”) in comparison with their friends.

#### Physical activity and sedentary behaviour

*Accelerometry* will be used to objectively assess physical activity and sedentary time. Women will be asked to wear a tri-axial accelerometer (GT3X+, Pensacola, Florida, USA) for 9 consecutive days, starting the same day they receive the monitor (e.g. participants who receive the accelerometer on Monday, will carry the device until Tuesday of the next week). The first and last day will be excluded from the analyses, accounting for a total of 7 days of registering. Participants will be instructed to wear the accelerometer during the whole day (24 h) on their lower back attached by an elastic belt. To prevent any damage to the devices, these will be taken off during water-based activities such as bathing or swimming. Time engaged in light, moderate, and moderate-vigorous intensity physical activity and sedentary time will be calculated.

The *Sedentary Behaviour Questionnaire* [29, 30] assesses the amount of time spent doing 11 behaviours. The 11 items will be completed separately for weekday and weekend. Response options are none, 15 min or less, 30 min, 1 h, 2 h, 3 h, 4 h, 5 h, or 6 h or more. The time spent on each behaviour will be converted into hours (e.g., a response of 15 min will be recorded as 0.25 h). For the total scores of sedentary behaviour, hours per day for each item will be summed separately for weekday and weekend. To obtain weekly estimates, weekday hours will be multiplied by 5 and weekend hours will be multiplied by 2 and these will be summed for total hours/week.

The short version of the *ALPHA Environmental questionnaire* [31] will be used to assess environmental perceptions about physical activity. The questionnaire will provide information about types of residences in the neighbourhood, distances to local facilities, walking or cycle infrastructure in the neighbourhood, cycling and walking network, neighbourhood safety, home and work/study environment mode of active travel.

#### Sleep quality

The *Pittsburgh Sleep Quality Index* [32] will be used to assess sleep quality and disturbances over a l-month time interval. Nineteen individual items generate seven “component” scores: subjective sleep quality, sleep latency, sleep duration, habitual sleep efficiency, sleep disturbances, use of sleeping medication, and daytime dysfunction. Each component yields a score ranging from 0 to 3, with 3 indicating the greatest dysfunction. The seven component scores are summed to provide a global sleep quality score (range 0 to 21) with higher scores indicating poorer sleep quality.

#### Quality of life, mental health and positive health

We will determine participants’ quality of life with the *Short-Form Health Survey 36* (SF-36) [33]. The SF-36 is a generic instrument for assessing health-related quality of life. It contains 36 items grouped into 8 dimensions: physical functioning, physical role, body pain, general health, vitality, social functioning, emotional role, and mental health. The scores range from 0 to 100 in every dimension, where higher scores indicate better health.

The *EQ-5D-5 L* consists of two parts. The first will be used to assess 5 dimensions of health related quality of life: mobility, self-care, usual activities, pain/discomfort and anxiety/depression, each of which is defined through five severity levels [34, 35]. The instrument therefore defines 3125 different health states from all the possible combinations of dimensions and levels of severity. EQ-5D-5 L health states may be converted into a single index value [34, 35]. This index ranges from 1 (best health status) to 0 (death). The second part consists of a 20-cm, vertical visual analogue scale (VAS), in the form of a thermometer, with endpoints of worst and best imaginable health status (scored 0 and 100, respectively) [34, 35]. This questionnaire will be administered at 3 different time points: at the beginning of the study, at 8 weeks and at 16 weeks, moreover it will be used to perform the cost-utility analysis.

The *Rosenberg Self-Esteem Scale* [36] is a 10-item scale to analyse the global self-esteem [37]. Each item is rated on a 4-point Likert scale, ranging from 1 = “strongly disagree” to 4 = “strongly agree”. The score ranges from 10 to 40, and higher scores reflect greater self-esteem.

The *brief COPE* [38, 39] is a 28-item scale that assesses coping strategies. Participants will be asked to rate how often they use specific coping strategies on a 4-point Likert scale, from 0 = “I haven’t been doing this at all” to 3 = “I’ve been doing this a lot”. The 28 items are paired together describing 14 different coping strategies: active coping, planning, positive reframing, acceptance, humour, religion, use of emotional support, use of instrumental support, self-distraction, denial, venting, substance use, behavioural disengagement and self-blame. Score for each subscale ranges from 0 to 6, and higher score indicates more frequency of the particular coping strategy.

The *General Self-efficacy Scale* [40, 41] is a 10-item scale to analyze the global self-efficacy. Each item is rated on a 4-point Likert scale, ranging from 1 = “not all true“ to 4 = “exactly true”. The score ranges from 10 to 40 where higher scores reflect greater general self-efficacy.

The *Beck Depression Inventory-II* [42, 43] will be used to assess depression severity. It contains 21 items measuring depressive symptoms such as sadness, pessimism, suicidal thoughts or wishes, tiredness or fatigue, loss of energy, and loss of pleasure, among others. Each item is scored on a scale ranging from 0 to 3. The global score ranges from 0 to 63 with higher score indicating greater degree of depression. A score of ≤13, 14–19, 20–28, and ≥29 represents minimal, mild, moderate, and severe depressive symptoms, respectively

The *State Trait Anxiety Inventory* [44] consists of 2 scales for measuring state anxiety and trait anxiety. Each scale comprises 20 items with scores ranging from 1 to 4. The total score of each scale ranges from 20 to 80 and higher values indicate higher levels of anxiety.

Positive health will be assessed by means of the following questionnaires:

1. The *Trait Meta-Mood Scale* [45] is comprised of 3 subscales to assess emotional attention, emotional clarity and emotional repair. Each subscale comprises 8 items. Participants rate their responses using a 5-point Likert scale, with 1 = “strongly disagree” to 5 = “strongly agree”. The subscales score range from 8 to 40 and higher scores reflect greater emotional attention, clarity, and repair.
2. The *Positive and Negative Affect Schedule* [46, 47] is a 20-item questionnaire designed to measure positive and negative affect. The questionnaire includes 10 positive and 10 negative emotional states that should be answered on a 5-point Likert scale, from 1 = “very slightly or not at all” to 5 = “extremely”. The score ranges from 10 to 50 for both subscales (positive affect and negative affect), and higher scores reflect greater affective well-being.
3. The *Satisfaction with Life Scale* [48, 49] assesses the perceived global life satisfaction. It consists of 5 items with a 5-point Likert scale from 1 = “strongly disagree” to 5 = “strongly agree”. The score ranges from 5 to 25, and higher scores reflect greater life satisfaction.
4. The *Life Orientation Test Revised* [50, 51] is a 10-item scale that assesses participants’ expectations about their future and their general sense of optimism. Six items (three reverse-scored) are used to obtain the total optimism score. Each item is rated on a 5-point Likert scale ranging from 0 (“strongly disagree”) to 4 (“strongly agree”). The total score ranges from 0 to 24, with higher scores indicating higher levels of optimism. The remaining 4 items are filler questions.
5. The *Subjective Happiness Scale* [52, 53] is a 4-item scale that assesses the global subjective happiness. Each item is rated on a 7-point Likert scale (e.g., “In general, I consider myself”, from 1 = “Not a very happy person” to 7 = “A very happy person”). The total score is the mean of all the four items, ranging from 1 to 7, and higher score indicates greater subjective happiness.
6. The *10-item Connor-Davidson Resilience Scale* [54, 55] assesses resilience to stress, which is a construct refering to a dynamic process of positive adaptation to adverse changes in life circumstances [56, 57]. Each item ranges from 0 = “not true at all” to 4 = “true nearly all the time”. The total score range from 0 to 40, and higher scores indicate greater resilience.

#### Menopause symptoms

The *Blatt-Kupperman menopausal index* [58] consists of 11 items including sweating/hot flushes, palpitation, vertigo, headache, paraesthesia, formication, arthralgia, and myalgia (categorized as somatic symptoms), and fatigue, nervousness, and melancholia categorized as psychological symptoms. A scale ranging from 0 to 3 points is used to describe the severity of the complaints. The total score ranges from 0 to 63, calculated as the sum of all items by the weighting factor. Scores ranging from 0–6, 7–15, 16–30, and 30 are used to rate the degree of severity as none, mild, moderate, and severe, respectively.

The *Cervantes scale* [59] is a health-related quality of life questionnaire specific for the menopause. The scale has 31 items and covers 4 domains: menopause and health (15 items), psychological domain (9 items), sexuality (4 items), and couple relationship (3 items). Each item score ranging from 0 to 5. The global score range from 0 to 155 (from better to worse quality of life).

### Measurements procedures

The women will be cited three days to complete all the measurement protocol. The first day, participants will attend the primary care centre and complete the following assessments in the same order as presented here: socio-demographic and clinical information, blood pressure and resting heart rate, body composition and physical fitness. The participant will then receive the accelerometer and all the questionnaires to be completed at home and will be cited eight days later to return them. Additionally, the primary care centre will cite participants another day for the biochemical analysis.

### Statistical analysis

Baseline characteristics of the study participants will be presented as mean (standard deviation) for continuous variables and proportions for categorical variables.

Statistical analysis will be performed on the intention to treat population, thus including all randomized participants (in the groups to which they were randomly assigned) in the analysis. Missing data will be replaced using multiple imputations. The effects of the intervention on the primary and secondary outcomes will be assessed with repeated measures analysis of covariance adjusted for age and baseline values. The effect size (95 % confidence interval) and statistical significance will be reported for each study outcome with regards to the main group (between-subjects) time (within-subjects) and their interaction (group × time) effects. The statistical significance will be set at the conventional level of p<0.05. To account for potential estimations biases or inefficiency, sensitivity analyses will be undertaken using baseline observation carried forward imputation, and also the available case population (participants who actually received the treatment; i.e. no imputations).

*Ancillary analyses.* "Per-protocol" analyses will also be performed on participants with overall ≥75 % treatment compliance.

#### Cost-effectiveness analysis

A cost-utility analysis with a health system perspective will be conducted in line with the methodological recommendations suggested by Lopez-Bastida et al. [60], and the guidelines defined in the economic evaluation literature [19, 61].

The study will be conducted considering the costs and the health effects of the intervention. Only direct costs of the program will be identified and valued. As direct costs for both groups, the drugs consumption, primary care visits, and hospital admissions will be considered. In the intervention group, the time dedicated by sport professionals of the exercise program will also be considered. Their total remuneration will be calculated based on the collective agreement for these professionals. First, for each group (intervention and control) an average of cost and effectiveness (the mean QALYs gained) will be calculated. Secondly, the ICER [19] will be calculated. To analyse the uncertainty in model parameters and verify the robustness of the ICER, various deterministic and probabilistic sensitivity analyses will be held [62]. The probabilistic analysis will be carried out through bootstrapping non parametric methods with 1000 replications. The resulting 1000 ICER replications will be plotted on the cost-effectiveness plane and will be used to construct an acceptability curve [63]. The cost-effectiveness plane is a graphical way of presenting cost-effectiveness results, with the difference in costs on the vertical axis and the difference in health benefits on the horizontal axis. Since incremental costs and health benefits can both be either positive or negative, there are four possible combinations, which will be reflected in the four quadrants of the cost-effectiveness plane:

- Upper-left quadrant: intervention less effective and more costly than comparator.
- Lower-left quadrant: intervention less effective and less costly than comparator.
- Upper-right quadrant: intervention more effective and more costly than comparator.
- Lower-right quadrant: intervention more effective and less costly than comparator.

The acceptability curve represents the proportion of simulations in which the intervention was considered cost-effective over a range of values of the threshold cost- per-QALY [64]. All analyses will be conducted in Stata v.13 (Stata Corp, College Station, TX, USA).

## Discussion

This paper describes the protocol for a RCT that aims to determine the clinical and cost-effectiveness of a primary care-based exercise intervention in perimenopausa women. Physical activity during the menopause transition and post menopause period has been reported to improve mental health, prevent weight gain, increase bone mineral density and muscle mass and reduce the risks of other diseases (e.g. cancer, diabetes, heart disease) [5, 65]. Additionally, increasing evidence suggests that exercise is a safe and useful strategy for alleviating menopause symptoms [65], with no serious reported side-effects [65]. For all those reasons, policy makers should consider encouraging general practitioners to prescribe exercise programs in primary care settings in collaboration with exercise specialists [66]. In Spain, the 2012 national health survey [67] revealed that 82 % of the population visited primary health care centres, making this an ideal setting for undertaking exercise intervention programs [68]. Most interventions to increase physical activity in primary care have been cost-effective [68]. A review showed the effectiveness of at least 12 months of primary care-based interventions to promote physical activity [69]. However, most of the interventions involved advice or counselling given face to face, or by phone (or both), and few trials have investigated supervised exercise interventions [69]. Further exercise interventions are needed, with objective outcome measures [69]. This project will be a primary care-based RCT and its results may potentially be generalizable to similar primary care settings. The designed intervention is also feasible and non-expensive. If the present RCT proves to be effective, it can be easily transferred to other similar contexts. Additionally, we will analyse the association of physical activity, sedentary behaviour and physical fitness with the other variables registered and with pharmacological and health service costs. Consequently, the findings of the FLAMENCO Project will help the health care system to identify preventive strategies for primary prevention and health promotion as well as strategies to reduce health care requirements and cost.

## Abbreviations

CVD: Cardiovascular disease
GDP: Gross domestic product
ICER: Incremental cost effectiveness ratio
QALY: Quality adjusted life years
RCT: Randomized controlled trial
RPE: Rate of perceived exertion
SF-36: Short-form health survey 36
